# Comorbidities and survival in patients with chronic hypersensitivity pneumonitis

**DOI:** 10.1186/s12931-020-1283-8

**Published:** 2020-01-09

**Authors:** Julia Wälscher, Benjamin Gross, Julie Morisset, Kerri A. Johannson, Martina Vasakova, Jacques Bruhwyler, Michael Kreuter

**Affiliations:** 1 Centre for Interstitial and Rare Lung Diseases, Pneumology and Respiratory Critical Care Medicine, Thoraxklinik, University of Heidelberg, Röntgenstr. 1, D-69126 Heidelberg, Germany; 2 Centre Hospitalier de l’Universite de Montreal, Quebec, Canada; 3 Department of Medicine, University of Calgary, Medicine, Calgary, Canada; 4 Department of Respiratory Medicine, First Faculty of Medicine of Charles University, Thomayer Hospital, Prague, Czech Republic; 5 ECSOR Department of Biostatistics, Gembloux, Belgium; 6 German Center for Lung Research, Heidelberg, Germany

**Keywords:** Interstitial lung disease, Extrinsic allergic alveolitis, Comorbidities, Pulmonary fibrosis

## Abstract

**Introduction:**

Chronic Hypersensitivity Pneumonitis (cHP) is a fibrotic interstitial lung disease (ILD) resulting from repeated exposure to an offending antigen. Prognostication in cHP remains challenging, and the relationship between comorbidities and survival has yet to be characterized. The aim of this study was to describe the relationship between comorbid conditions and survival in patients with cHP.

**Methods:**

The prospective database from a tertiary referral centre for ILD was reviewed for patient-reported comorbidities, their frequency, and relationship with survival in cHP patients. Comorbidities were assessed by direct questioning of the patient at the baseline visit and by a standardized questionnaire for the diagnosis of interstitial lung diseases. During the follow-up examinations, patients were asked about newly diagnosed comorbidities.

**Results:**

Two hundred eleven patients with cHP were identified (mean age 63 years, 53% male, mean FVC 73%), with mean follow-up of 32 months. The mean number of comorbidities was 3 (10% had 0, 59% 1–3 and 31% ≥4 comorbidities). Most frequent comorbidities groups were cardiovascular (65%) and respiratory (26%), most common comorbidities were hypertension (56%), gastro-esophageal reflux disease (GERD) (24%), diabetes (20%) and coronary heart disease (18%). In general, deceased patients had more comorbidities than survivors (*p* = 0.005), yet there was no association between the absolute number of comorbidities and survival. Pulmonary hypertension (30.8% versus 5.7%, *p* = 0.001;), diastolic dysfunction (26.9% versus 6.4%, *p* = 0.004) and cerebrovascular disease were more frequent in non-survivors (23.1% versus 7.6%, *p* = 0.026). Lung cancer was not observed, and neither GERD nor antacid drugs were associated with outcome (*p* = 0.357 and *p* = 0.961, respectively).

**Conclusions:**

Comorbidities are common in cHP are associated with survival. Further work should determine whether interventions for these specific comorbidities can positively affect survival.

## Introduction

Chronic hypersensitivity pneumonitis (cHP) is an interstitial lung disease, where sensitization to an inhaled antigen leads to inflammation and subsequent fibrosis in the lung parenchyma [1, 2]. cHP is a complex disease that can be challenging to diagnose and manage, even in experienced multidisciplinary teams [3, 4]. The simultaneous presence of comorbid conditions may further complicate the diagnosis by negating the potential for invasive diagnostic procedures or presenting overlapping features such as in smoking related emphysema [5]. Besides presenting challenges in diagnosis, comorbidities may also influence prognosis. The long-term prognosis of patients with cHP is associated with the extent of radiologic fibrosis, a lack of identified exposure, older age, lower forced vital capacity (FVC) at baseline and positive smoking history [6, 7]. While the relationship between comorbidities and survival has been characterized in patients with idiopathic pulmonary fibrosis (IPF) [8, 9], it is unknown how comorbid conditions may affect prognosis in patients with cHP [10]. The aim of this study was to determine if specific comorbidities and/or the overall burden of comorbidities are associated with survival in patients with cHP.

## Methods

### Study population

The database of our tertiary referral center for interstitial lung diseases (ILD) was reviewed for patient-specific comorbidities, their frequency and relationship with survival in cHP. The study included patients diagnosed between June 1995 and November 2017. All clinical diagnoses were established via multidisciplinary team discussion consisting of ILD-experienced clinicians, radiologists and pathologists. All patients underwent HRCT scan of the chest and many underwent histopathological sampling (79%).

Multidisciplinary diagnosis of cHP was established based on clinical history, bronchoalveolar lavage (BAL) fluid analysis, either patterns on high-resolution CT (HRCT) plus identification of a plausible exposure and/or histopathological findings consistent with cHP in lung biopsy samples consistent with a recent delphi survey on cHP [11]. For diagnostic purposes we used the algorithm described therein, in which exposure, typical characteristics on HRCT (a combination of mosaic attenuation, ground-glass and normal lung or combination of mosaic attenuation and radiologicals signs of fibrosis), the lymphocyte percentage in BAL and typical characteristics on histology (chronic bronchiolocentric inflammation, poorly formed non-necrotizing granulomas, giant cells, airway centered interstitial fibrosis and absence of alternative diagnosis.) are taken into account.

The following data were collected at the time of diagnosis: demographics including age, sex, smoking history (and pack years), occupation, family history of ILD, pulmonary function tests (absolute and % predicted of forced vital capacity (FVC), forced expiration volume in 1 s (FEV_1_), FEV_1_/FVC ratio and diffusing capacity of the lung for carbon monoxide (DLCO)), diagnostic procedures, histological pattern on biopsy where available, BAL analysis and specific antigen exposure.

The Ethics Committee of the University of Heidelberg approved this retrospective study. (S-318/2013).

### Comorbidities

Comorbidities were assessed by direct questioning of the patient at the baseline visit and by a standardized questionnaire for the diagnosis of ILD [12]. In addition, comorbidities were assessed in medical reports and recorded as dichotomous variables as present or absent. At each follow-up (every 9–12 months), patients were questioned about newly diagnosed comorbidities. The time interval between the diagnosis of cHP and the diagnosis of the individual comorbidity was determined. Also current medications were listed.

The following comorbidities were assessed: Asthma, pulmonary hypertension, obstructive sleep apnea (OSA), chronic obstructive pulmonary disease (COPD), diastolic dysfunction, hypertension, renal insufficiency, liver failure, thyroid disease, anemia, osteoporosis, coronary heart disease, diabetes mellitus, peripheral arterial occlusive disease (PAOD), cerebrovascular disease, thromboembolic events, atrial fibrillation (AF), gastroesophageal reflux disease (GERD), inflammatory rheumatic systemic diseases, fibromyalgia, Raynaud’s syndrome, mental illness, migraine, lung cancer, other malignant diseases.

## Statistical analysis

Descriptive statistics were used to characterize the patient population. Continuous variables were characterized by the N, n with missing data, mean, standard deviation (SD), median, minimum and maximum. Discrete variables were characterized by the N, n for each category and corresponding percentages. Frequencies of discrete variables were compared between patients using Fisher’s exact tests. Median survival times with 95% confidence intervals (95% CI), were determined using Kaplan-Meier curves. The log-rank test was used to compare different survival curves by patient categories. Variables associated with survival were identified using multivariate Cox proportional hazards regression models.

IBM SPSS Statistics (Version 21.0) and StatXact (Version 6.0) were used for the statistical analyses. Missing values were not replaced. By general convention, *p* values lower than 0.05 were considered statistically significant.

## Results

### Study population

There were 211 cHP patients identified with baseline characteristics shown in Table 1. Mean (± SD) age was 63.0 ± 13.3 years at diagnosis and 112 (53%) were male (*n* = 112). Median FVC was 71% ± 21 predicted and DLCO was 44% ± 14 predicted. Median follow-up of the cohort was 13.8 years with standard error (SE) of 12.1 months.

**Table 1:** Patient characteristics at baseline

Characteristics	n/(%)	Mean	SD
Age at first diagnosis (years)	211	62,96	13,3
Gender (n)			
Males	112 (53,1)		
Females	99 (46,9)		
Smoking status	210		
Non-smokers	106 (50,2)		
Active smokers	9 (4,3)		
Former smokers	95 (45,2)		
Pack-years	206	11,01	16,2
Family history for lung diseases			
Family history for ILD	8 (3,8)		
Pulmonary function			
FVC (% predicted)	209	72,68	21,20
FVC (L)	207	2,41	0,92
FEV1 (% predicted)	210	76,82	20,12
FEV1/FVC%	209	81,94	8,52
TLC (L)	205	4,31	1,19
TLC (% predicted)	207	74,14	15,92
DLCO-SB (% predicted)	185	44,10	13,92
6MWD (m)*	164	373,43	112,14
Modified GAP stage [13]			
I	89,1		
II	8,5		
III	2,4		

*6 MWT = 6 minutes walking test

### Comorbidities and survival

The mean number of comorbidities per patient was 2.78 ± 2.03 (0–13). Twenty-two patients (10.4%) had no comorbidities, 123 (58%) had between 1 and 3 and 66 (31%) had ≥4 comorbidities. The most frequent organ groups of comorbidities were cardiovascular (65%) and respiratory (26%). Most common comorbidities were arterial hypertension (56%), GERD (24%), diabetes (20%) and coronary heart disease (18%). In 11.4% there was a history of cancer other than lung cancer prior to the diagnosis of ILD, there was no prior history of lung cancer. Survival was assessed and reasons for death categorized into acute exacerbations, infections, right heart failure, progressive fibrosis, cardiovascular and unknown. The causes of death are shown in Table 3. During follow up there were no incident cases of lung cancer identified. The prevalence of comorbidities in this cohort is shown in Table 2.

**Table 2:** Prevalence of comorbidities

	*N* = 211, Frequency (%)
Anemia	8, (3.8)
Asthma	11 (5.2)
Atopy	38 (18)
Cerebrovascular disease	9 (9)
Coronary heart disease	37 (17.5)
Chronic obstructive pulmonary disease	9 (4.3)
Depression	18 (8.5)
Diabetes mellitus	43 (20.4)
Diastolic dysfunction	21 (10)
Hypertension	117 (55.5)
Gastroesophageal reflux disease	50 (23.7)
Liver disease	9 (4.3)
Lung cancer	0
Other cancers	24 (11.4)
Adrenocortical cancer	1 (0.47)
Anal cancer	1 (0.47)
Basalioma	1 (0.47)
Bladder cancer	2 (0.95)
Mammary cancer	4 (1.90)
Colon cancer	1 (0.47)
cancer of unknown primary	1 (0.47)
Liposarcoma	1 (0.47)
Malignant Melanoma	2 (0.95)
Myelodysplastic syndroma	1 (0.47)
Prostate cancer	6 (2.84)
renal cell cancer	2 (0.95)
Unknown cancer	2 (0.95)
Peripheral artery occlusive disease	8 (3.8)
Pulmonary hypertension	20 (9.5)
Renal insufficiency	7 (3.3)
Rheumatic disease	20 (9.5)
Obstructive sleep apnea	19 (9.0)
Osteoporosis	26 (12.3)
Other cardiovascular diseases	35 (16.6)
Thromboembolic disorders	10 (4.7)
Thyroid disease	31 (14.7)
Vasculitis	0

**Table 3:** Survival

Characteristics	N	%	Mean	Median	range	SD
Survival status	211					
Alive	157	74,4				
Unknown	28	13,3				
Deceased	26	12,3				
Survival time (month)	194		32,41	15,65	0–218,22	43,67
Death reason	26	100				
Exacerbation	7	26,9				
Sequelae (right heart failure, progressive fibsosis, e.t.c)	4	15,4				
Cardiovascular	5	19,2				
Unknown	10	38,5				

**Fig. 1:**
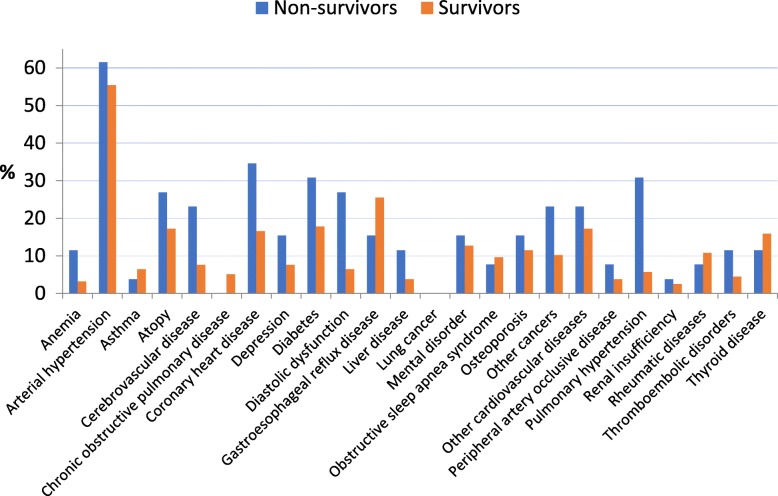
Prevalence of individual comorbidities in relation to survival status (survivor vs. non-survivor)

There was no association between overall number of comorbidities and survival. When the prevalence of individual comorbidities in relation to survival status (survivor vs. non-survivor) was considered, pulmonary hypertension (30.8% vs. 5.7%) (*p* = 0.001) and cerebrovascular disease (23.1% versus 7.6%) (*p* = 0.026;) were more prevalent in deceased patients (Fig. 1).

**Fig. 2:**
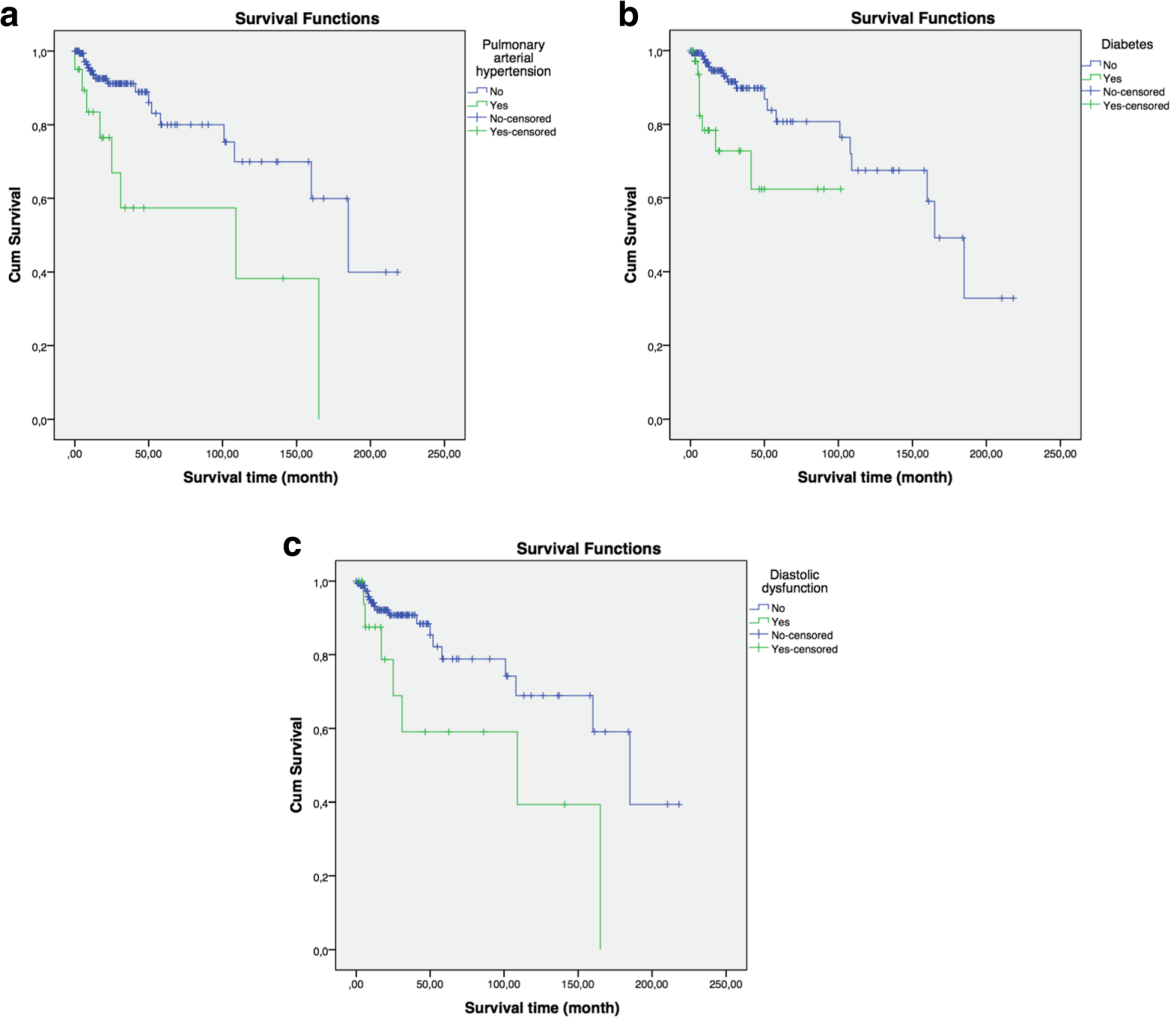
**a**-**c**: Kaplan-Meier survival curves for patients with or without (*p* = 0.002) pulmonary arterial hypertension, for patients with or without (*p* = 0,002) diabetes and diastolic dysfunction (*p* = 0,016)

Survival duration was significantly and negatively associated with pulmonary hypertension (*p* = 0.002; log rank test), diabetes (*p* = 0.002; log rank test) and diastolic dysfunction (*p* = 0.016; log rank test), as shown in Fig. 2a-c. There was no colinearity and only 2 patients who died had those three comorbidities at the same time.

In multivariate analysis considering all individual comorbidities, history of hypertension was associated with a worse survival (Hazard ratio [HR] 3.6; 95% CI =1.037–12.444, *p* = 0.044), while thromboembolic disorders was associated with an improved survival (HR 0.1; 95% CI = 0.021–0.809, *p* = 0.029).

In patients with pulmonary hypertension depending on an UIP pattern in CT there was a significant association. (*p* = 0,044). A total of 20 patients had pulmonary hypertension in our patient population, of which 15 had no UIP pattern, 4 had a typical UIP pattern and 1 patient had a possible UIP pattern. There was also a significant correlation in comorbidity GERD. (*p* = 0,038). Of the 50 patients with GERD, 44 had no UIP pattern, none had a typical UIP pattern,and 6 had a possible UIP pattern. All other comorbidities showed no significant relationship to the HRCT pattern.

## Discussion

Here we describe an association between distinct comorbid conditions and survival in chronic HP. In this cohort, a history of pulmonary hypertension, diabetes or diastolic dysfunction were associated with worse survival. While in IPF a clear association between the frequency of comorbidities and survival is established [8], this was not the case in this report. Reasons are unclear but might be associated to an even older population in IPF than in HP or due to other factors like physical activity and a lower smoking prevalence in cHP than in IPF.

These data are consistent with prior findings. Koschel et al. described that pulmonary hypertension which was present in 19% of the patients, was associated with a higher risk of death in a cohort of 120 patients with chronic HP. [14] Yet, while this and other data clearly indicate the negative impact of PH in cHP, there is a paucity of data to guide PH screening. Furthermore, there are no data to inform whether drug treatment of cHP may favorably influence pulmonary hypertension and whether PAH drugs improve survival in HP associated PH [8, 15]. Yet, no significant benefit has been demonstrated in patients with IPF treated with PAH therapies or a combination of the antifibrotic drug nintedanib and sildenafil [16–19]. However, it is certainly difficult to distinguish whether pulmonary hypertension is a comorbidity or a complication of pulmonary fibrosis. However, pulmonary hypertension reflects the severity of the disease. The data on diastolic dysfunction seem to be quite interesting compared to IPF, where some recent data report a different connection with death [8].

Little is known about the relationship between diabetes and cHP. Comparable to IPF, 20% of our patients had diabetes [20]. Whether diabetes might be considered a potential risk factor for HP progression is unclear. Recent data suggest a relationship between diabetes and IPF [21] and hypothetically steroid treatment associated hyperglycemia might influence the severity of IPF [22]. While steroid treatment is not a primary therapy for IPF, corticosteroids are still the most commonly prescribed treatment in cHP [23]. Thus, it cannot be excluded that an association between steroid treatment and negative outcomes in relation to diabetes may exist. Yet, further translational research is needed in this regard. It is also to be discussed whether antidiabetic treatment strategies could improve the course of cHP. However, in a recent study a prognostic role of metformin in patients with IPF, could not have been established [24].

Little is known about the impact of diastolic dysfunction in patients with cHP. In a smaller study of IPF patients, it was shown that patients with idiopathic pulmonary fibrosis have an early impairment of left ventricular diastolic function and it was demonstrated that patients with clinically stable IPF exhibit not only RV diastolic and systolic dysfunction but also impaired LV diastolic filling [25]. Kreuter et al. reported that diastolic dysfunction has a favorable effect on survival in IPF [8].

In chronic lung disease, thromboembolic events are frequent, especially during episodes of acute worsening. In IPF, thromboembolic events are associated with an unfavorable outcome [26]. Yet, in our cohort, thromboembolic events were associated with a more favorable outcome. These findings should be interpreted with caution given the small numbers, but warrant further investigation. We can also speculate that thromboembolism causing deterioration in cHP patients is treatable and reversible trait compared to other reasons, for instance left ventricle dysfunction or acute exacerbation of cHP.

GERD has been proposed as a risk factor for the progression of IPF while data on antiacid treatment are conflicting. Similar to a recent report in IPF [8] we here show that GERD is frequent in cHP.

This study has several limitations. This is a retrospective single centre cohort and our findings may not be generalizable. Comorbidities may have been misclassified, although efforts were made to verify patient-reported comorbidities using chart review. We could not account for patients taking disease-specific therapies for the listed comorbidities, and how they may impact clinical outcomes over time. Outcomes may also have been biased by other factors such as treatment effects on cHP, subtypes of cHP, age and functional limitations, however all cases were classified as chronic, fibrotic HP by an experienced multidisciplinary team.

In conclusion, we here report on an association between distinct comorbidities and survival in patients with cHP. Number of comorbidities may not influence prognosis of cHP patients, on the contrary to IPF. However, there appears to be a clinical meaningful effect of cardiovascular diseases on survival in cHP which highlights importance of identification and management of comorbidities in patients with cHP. Further studies are needed to fully clarify the importance of comorbidities on prognosis in patients diagnosed with cHP, and to determine whether specific treatments may positively impact outcomes in this patient population.

## Data Availability

The datasets used and/or analysed during the current study are available from the corresponding author on reasonable request.
